# Venetoclax for the treatment of translocation (11;14) AL amyloidosis

**DOI:** 10.1038/s41408-020-0321-6

**Published:** 2020-05-11

**Authors:** M. Hasib Sidiqi, Abdullah S. Al Saleh, Nelson Leung, Dragan Jevremovic, Mohammed A. Aljama, Wilson I. Gonsalves, Francis K. Buadi, Taxiarchis V. Kourelis, Rahma Warsame, Eli Muchtar, Miriam A. Hobbs, Martha Q. Lacy, David Dingli, Ronald S. Go, Suzanne R. Hayman, S. Vincent Rajkumar, Angela Dispenzieri, Morie A. Gertz, Shaji K. Kumar, Rafael Fonseca, Prashant Kapoor

**Affiliations:** 1 Division of Hematology, Department of Medicine, Mayo Clinic Rochester, Rochester, MN USA; 2 Department of Haematology, Fiona Stanley Hospital, Perth, Western Australia Australia; 3 Division of Nephrology, Department of Medicine, Mayo Clinic, Rochester, MN USA; 4 Division of Hematopathology, Mayo Clinic Rochester, Rochester, MN USA; 5 Juravinski Cancer Centre, McMaster University, Hamilton, ON Canada; 6 Division of Hematology and Oncology, Department of Medicine, Mayo Clinic Phoenix, Phoenix, AZ USA

**Keywords:** Myeloma, Targeted therapies

Dear Editor,

Systemic light chain (AL) amyloidosis is a clonal plasma cell disorder characterized by the deposition of immunoglobulin light chain-associated amyloid material in various organs^1^. While therapies such as high-dose melphalan followed by autologous stem cell transplantation (ASCT) can result in deep and durable responses^2^, only approximately a quarter of newly diagnosed patients are deemed transplant eligible^1,3^. Even with successful initial therapy, relapses occur, and not infrequently the response is of insufficient depth to protect against further amyloid deposits in vital organs. Alternative therapeutic options are required for such patients.

Venetoclax is an oral, small-molecule B cell lymphoma 2 (BCL-2) inhibitor that induces cellular apoptosis^4^, with encouraging activity in multiple myeloma (MM), particularly in patients whose clonal plasma cells harbor t(11;14) and/or overexpress BCL-2 (refs. ^5,6^). In preclinical models, venetoclax demonstrates synergy in combination with bortezomib against MM cell lines^7^. Approximately 50% of patients with AL amyloidosis have t(11;14), making venetoclax a suitable agent to treat this rare disorder^8–10^. Venetoclax could induce a complete response (CR) in a patient with AL amyloidosis whose disease markers plateaued on bortezomib, cyclophosphamide, and dexamethasone (VCD)^11^. A case series presented in an abstract form, involved seven patients with relapsed/refractory AL amyloidosis (RRAL) with cardiac involvement who were treated with venetoclax^12^. Two of four patients who were treated with at least two cycles of venetoclax achieved a CR and had t(11;14). We report a larger cohort demonstrating efficacy and safety of venetoclax in RRAL.

We conducted a review of records of all patients with RRAL, treated with venetoclax between January 2017 and May 2019 at Mayo Clinic. Organ involvement and response was assessed according to the Consensus Criteria^13,14^. Patients with normal light chain levels at time of initiation of venetoclax were considered inevaluable for hematologic response. The patients were risk stratified according to the 2012 Mayo staging system^15^.

We identified 12 patients who were treated with venetoclax for RRAL amyloidosis, with patient characteristics listed in Table 1. The median age was 64 years (range 52–76) and 75% (*n* = 9) were males. The median number of prior lines of therapy was 2 (range 1–4). Previous therapy exposures included proteasome inhibitors (100%), oral alkylators (92%), or high-dose melphalan with autologous stem cell rescue (25%), immunomodulatory drugs (25%) and anti-CD 38 antibodies, daratumumab (33%). Most common organs involved were renal (75%) followed by heart (50%), neurological (25%), and gastrointestinal (17%). The t(11;14) signature was detected on interphase fluorescence in situ hybridization (FISH) studies of the marrow in 11 of 12 patients. Of these 11 patients with t(11;14), BCL2 expression by immunohistochemistry was assessed in 5 patients, all of whom exhibited strong expression. Venetoclax was used alone or in combination with dexamethasone in seven (58%) patients. The remainder received venetoclax in a triplet or quadruplet combination that invariably included bortezomib and dexamethasone. The quadruplets administered to two patients incorporated lenalidomide and cyclophosphamide, respectively, to venetoclax, bortezomib, and dexamethasone backbone.

**Table 1:** Characteristics of each patient in the cohort.

Case	Age	Organ involvement	Mayo Stage 2012^a^	Prior lines of therapy	Venetoclax regimen	Dose (mg)	Hematologic tesponse	Duration of therapy (months)	Continuing therapy/reason for stopping
1	60	R	2	3	Ven	800	NE^b^	2.7	Y
2	68	R	3	2	Ven	400	VGPR	9.4	Y
3	68	H, R	2	2	VenBd	400	VGPR	27	Y
4	52	R	1	2	VenBd	400	NE^b^	19.1	N/G
5	58	H	3	3	VenBd	800	CR	5.1	N/G
6	64	R, N	1	2	VenBRd	800	CR^b^	3.4	N/T
7	55	H, N, GI	1	2	Ven	400	CR	11.7	Y
8	64	H, R	3	1	VenBCd	800	NR	5.3	N/L
9	76	H, R, GI, N	1	2	Ven-d	800	VGPR	2.5	N/T
10	63	H	1	3	Ven	800	NE	15.6	Y
11	75	R	1	1	Ven-d	800	CR	2	Y
12	72	R	1	4	Ven	400	NE	1.1	Y

*H* heart, *R* renal, *N* neurological, *GI* gastrointestinal, *Y* yes, *N* no, *MRD* minimal residual disease, *CR* complete response, *VGPR* very good partial response, *NE* not evaluable, *NR* no response, *Ven* venetoclax, *Bd* bortezomib and dexamethasone, *BRd* bortezomib, lenalidomide and dexamethasone, *d* dexamethasone, *BCd*, bortezomib, cyclophosphamide and dexamethasone, *G* goal achieved, *T* toxicity, *L* lack of response.

^a^Stage at the time of venetoclax initiation.

^b^MRD negative.

**Fig. 1:**
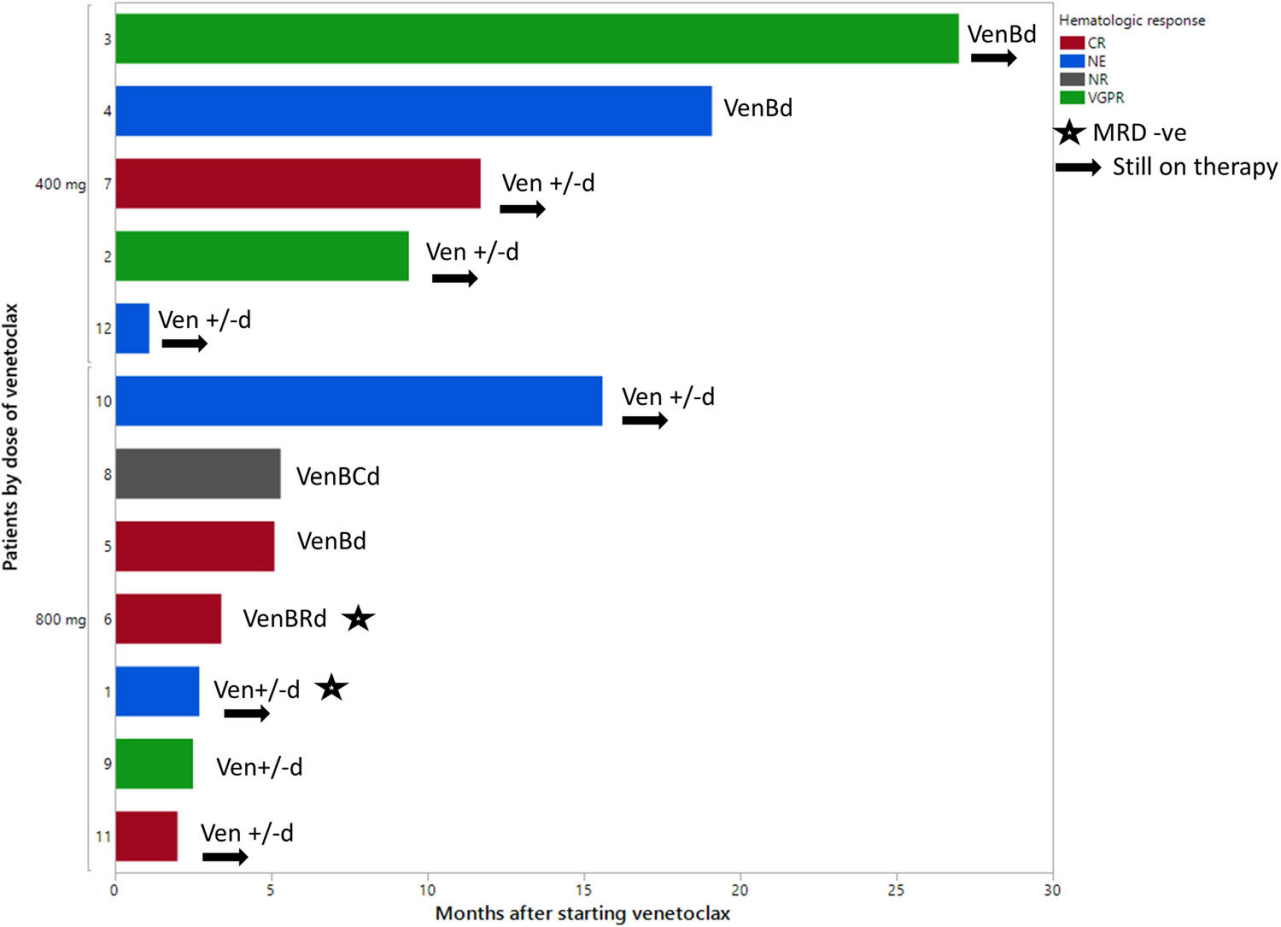
Response to venetoclax. Case numbers are displayed on the *Y-*axis. CR complete response, MRD minimal residual disease, NE not evaluable, NR no response, VGPR very good partial response, Ven venetoclax, d dexamethasone, VenBd venetoclax, bortezomib, and dexamethasone, VenBRd venetoclax, bortezomib, lenalidomide and dexamethasone, VenBCd venetoclax, bortezomib, cyclophosphamide and dexamethasone.

The dose of venetoclax used was 800 mg daily in 7 (58%) and 400 mg daily in 5 (42%) patients (Fig. 1). A dose ramp up to the final dose level was utilized in 8 (67%) patients. The median follow-up for the cohort was 11.5 months (95% confidence interval(CI): 2–21 months). The median duration of therapy was 5 months (range 1–27 months) and at last follow-up, 7 (58%) patients remained on venetoclax. Of eight patients who were evaluable for a hematologic response, four achieved a complete response (CR) (one patient with CR had undetectable minimal residual disease (MRD)), three achieved a very good partial response (VGPR), and one did not respond to therapy (overall response rate 88%) (Fig. 1). Four patients were inevaluable for hematologic response and one was found to be MRD negative on bone marrow assessment at 6 months after commencing venetoclax therapy. Of the five patients with strong BCL2 expression, three were evaluable for hematologic response and all three achieved CR (one attained MRD negative CR). The median time-to-best hematologic response was 3.4 months (range 1.6–8.4 months). At last follow-up, one of four patients with cardiac involvement achieved a cardiac response 3 months after initiation of venetoclax. Two of six evaluable patients with renal involvement achieved a renal response at 10 and 16 months post initiation of venetoclax, respectively.

None of the patients experienced tumor lysis syndrome and five have discontinued therapy. The reasons for the discontinuation included cytopenias (*n* = 1), dyspnea (*n* = 1), failure to respond (*n* = 1), and attainment of desired response (*n* = 2). The two patients who had stopped therapy after attaining a response did so at 19 months (renal response) and 5 months (hematologic CR), respectively.

Gastrointestinal side effects were reported in six patients (mild, with predominantly loose stools). One patient developed an upper respiratory tract infection (URTI) 3 weeks after starting venetoclax that only required supportive management and oral levofloxacin. Another patient developed an URTI a month after starting treatment and also pneumonia nearly a year later, which was uncomplicated and resolved with oral cefuroxime. At last follow-up, two patients have progressed at 4 and 5 months post initiation of venetoclax therapy, respectively, and none have died.

Our study shows that venetoclax is a generally well-tolerated and efficacious agent in patients with RRAL amyloidosis. It can effectively induce both hematologic and organ responses as a single agent or in combination with other agents. In our cohort, four patients have received venetoclax therapy for >12 months, of whom three are continuing on the drug, suggesting durability of response and tolerability of the agent. The patient who discontinued therapy did so after achieving a renal response and remains in remission. Notably, our study was almost exclusively in AL amyloidosis patients with presence of t(11;14). The single patient in whom the t(11;14) status was not available did not respond to therapy. Although venetoclax was used as a single agent or in combination with dexamethasone in the majority of our cohort, some patients did receive venetoclax as a component of a triplet or quadruplet.

Responses were seen in both subsets of patients receiving venetoclax as a single/doublet and those receiving it in other combinations. Given the small sample size and the retrospective nature of the current study, it is not possible to accurately ascertain the impact of single versus combination venetoclax-based therapies. Data on efficacy of venetoclax in early clinical trials (phase I/II) in myeloma were encouraging, with higher response rates seen in patients with t(11;14)^5,6^. However, in an interim analysis of an ongoing phase III study of venetoclax in combination with bortezomib and dexamethasone for relapsed or refractory myeloma (BELLINI trial, NCT02755597), safety concerns were raised as a result of increased number of deaths from both infectious and non-infectious causes in the venetoclax plus bortezomib and dexamethasone arm compared to the control arm of bortezomib and dexamethasone, despite a remarkable improvement in the progression-free survival. Despite these surprising findings, the subset of patients with t(11;14) MM, interestingly, demonstrated a trend towards improved survival in this trial. Patients with AL amyloidosis often have significant organ involvement (cardiac and kidney) and therefore these safety signals need to be carefully considered as prospective trials using venetoclax in this patient population are conducted (NCT03000660). Notably, in our cohort no deaths have been observed so far. Albeit a case series, with a small sample size, our study suggests high efficacy and good tolerability of venetoclax monotherapy and combination therapy in patients with RRAL amyloidosis who harbor t(11;14).
